# Supplementary material for: Temperature Restriction in Entomopathogenic Bacteria
Source: Front Microbiol. 2020 Sep 30;11:548800. doi: 10.3389/fmicb.2020.548800 (PMC7554251; doi:10.3389/fmicb.2020.548800)
Supplement: Supplementary file 1 [file Data_Sheet_1.PDF]

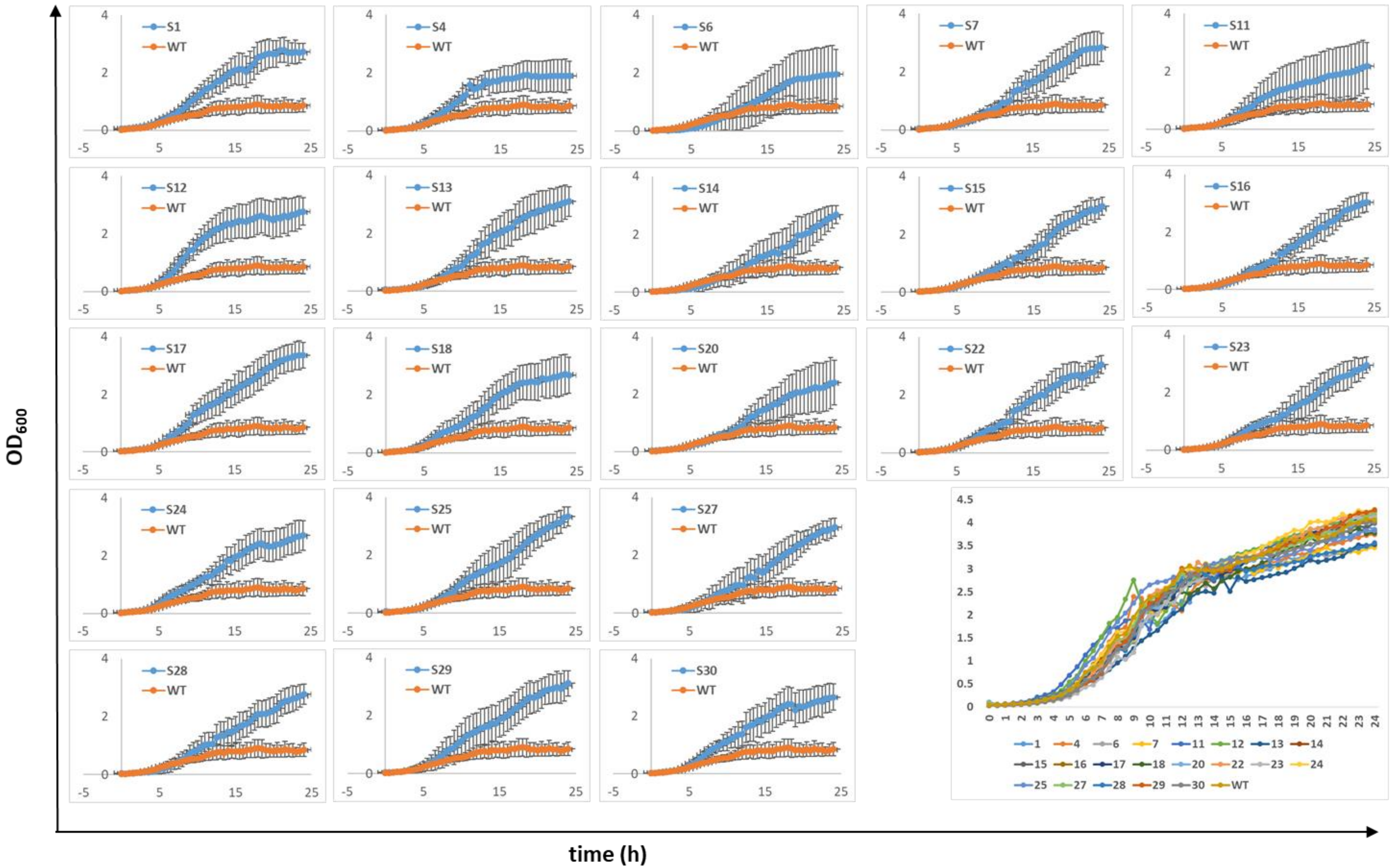

Supplementary Figure 1. Growth of isolates carrying a mutation in *trlG,* in liquid LB at 36 °˚C (individual panels) and at 28 °C (bottom right) in comparison to the WT. The error bars in the individual panels indicate the standard error from three independent experiments.
