# Supplementary material for: Temperature Restriction in Entomopathogenic Bacteria
Source: Front Microbiol. 2020 Sep 30;11:548800. doi: 10.3389/fmicb.2020.548800 (PMC7554251; doi:10.3389/fmicb.2020.548800)
Supplement: Supplementary file 2 [file Data_Sheet_2.PDF]

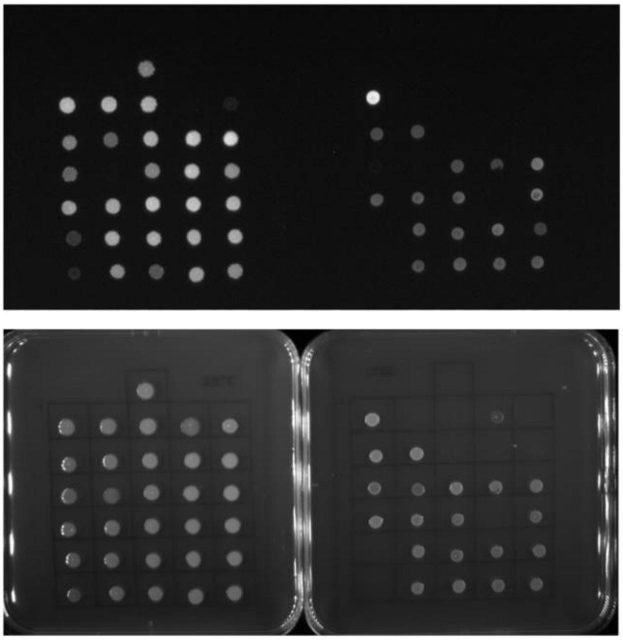

Supplementary Figure 2. Luminescence of the WT and the 30 sequenced tolerant clones shown in Figure 2A at 28 ˚C and 36 ˚C imaged using the GENESys SYNGENE imager (Top). At the bottom is the corresponding image of the plates taken with white light as a reference.
