# Supplementary material for: Temperature Restriction in Entomopathogenic Bacteria
Source: Front Microbiol. 2020 Sep 30;11:548800. doi: 10.3389/fmicb.2020.548800 (PMC7554251; doi:10.3389/fmicb.2020.548800)
Supplement: Supplementary file 4 [file Data_Sheet_4.PDF]

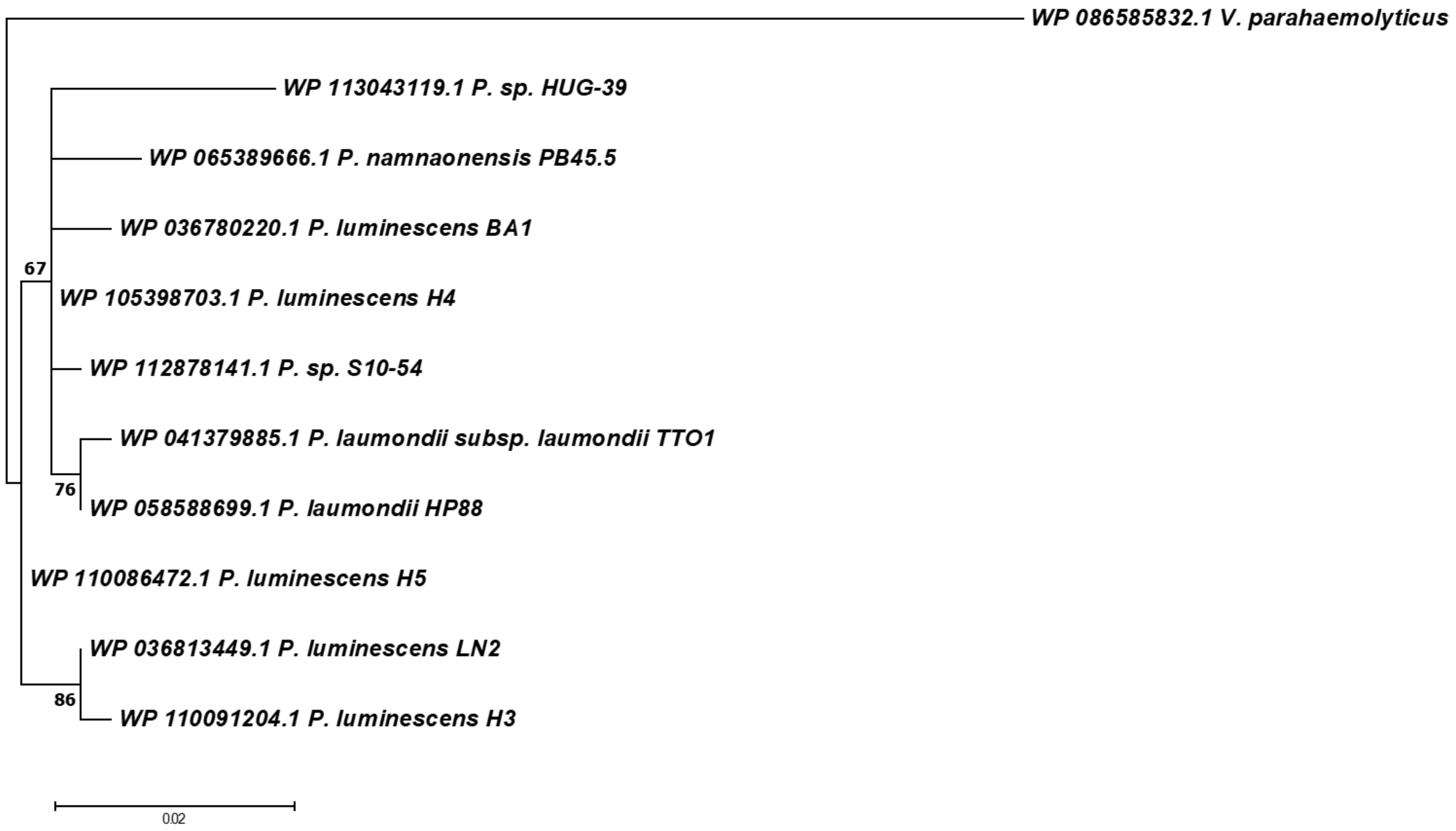

Supplementary Figure 4. Maximum likelihood tree demonstrating the phylogenetic relationship of the TrlG proteins encoded by the genomes of *Photorhabdus* bacteria; protein identifiers are RefSeq accession numbers. The protein encoded by *Vibrio parahaemolyticus* was included as an outgroup. Numbers at nodes show bootstrap support (%).
